# Effects of combined warmed preoperative forced-air and warmed perioperative intravenous fluids on maternal temperature during cesarean section: a prospective, randomized, controlled clinical trial

**DOI:** 10.1186/s12871-020-00970-7

**Published:** 2020-02-26

**Authors:** Ting-ting Ni, Zhen-feng Zhou, Bo He, Qing-he Zhou

**Affiliations:** 1 Department of Anesthesiology, NO.7 Hospital of Ningbo, Ningbo, Zhejiang Province China; 2 Department of Anesthesiology, People’s Hospital of Zhejiang Provincial (People’s Hospital of Hangzhou Medicine College), Hangzhou, Zhejiang Province China; 3 Department of Gynecology, NO.7 Hospital of Ningbo, Ningbo, Zhejiang Province China; 4 Department of Anesthesiology, The Affiliated Hospital of Jiaxing University, Jiaxing, Zhejiang Province China

**Keywords:** Cesarean section, Spinal anesthesia, Warming

## Abstract

**Background:**

Preventing the frequent perioperative hypothermia incidents that occur during elective caesarean deliveries would be beneficial. This trial aimed at evaluating the effect of preoperative forced-air warming alongside perioperative intravenous fluid warming in women undergoing cesarean sections under spinal anesthesia.

**Methods:**

We randomly allocated 135 women undergoing elective cesarean deliveries to either the intervention group (preoperative forced-air and intravenous fluid warming, *n* = 69) or the control group (no active warming, *n* = 66). The primary outcome measure was the core temperature change between groups from baseline to the end of the surgical procedure. Secondary outcomes included thermal comfort scores, the incidences of shivering and hypothermia (< 36 °C), the core temperature on arrival at the post-anesthesia care unit, neonatal axillary temperature at birth, and Apgar scores.

**Results:**

Two-way repeated measures ANOVA revealed significantly different core temperature changes (from the pre-spinal temperature to that at the end of the procedure) between groups (*F* = 13.022, *P* < 0.001). The thermal comfort scores were also higher in the intervention group than in the control group (*F* = 9.847, *P* = 0.002). The overall incidence of perioperative hypothermia was significantly lower in the intervention group than in the control group (20.6% vs. 51.6%, *P* < 0.0001).

**Conclusions:**

Warming preoperative forced-air and perioperative intravenous fluids may prevent maternal hypothermia, reduce maternal shivering, and improve maternal thermal comfort for patients undergoing cesarean sections under spinal anesthesia.

**Trial registration:**

The study was registered with the Chinese Clinical Trial Registry (registration number: ChiCTR1800019117) on October26, 2018.

## Background

Neuraxial (spinal, epidural, or combined spinal–epidural techniques) anesthesia is the preferred anesthetic technique for cesarean deliveries. Perioperative hypothermia is a commonly reported side effect of regional anesthesia affecting up to 60% of patients undergoing cesarean deliveries under spinal anesthesia [1–4]. The hypothermia can cause numerous complications including postoperative wound infections, increased blood loss and transfusion requirements, myocardial ischemia, high risk of coagulopathy, shivering, increased hospital stay, and patient discomfort [5–12]. Neonatal outcomes such as birth temperature and Apgar scores have also been linked to maternal temperature [13, 14].

Perioperative hypothermia under spinal anesthesia has different etiologies, but mostly it is caused by spinal anesthesia altering thermoregulation and reducing the threshold for vasoconstriction and shivering [15]. Neuraxial anesthesia decreases the thermoregulatory vasoconstriction below the sensory blockade level, leading to heat loss by redistribution of heat from the core to the periphery [16]. The core-to-peripheral redistribution of body heat is difficult to treat, but it should be preventable by prewarming the periphery compartment [17]. Prewarming increases the heat content in the periphery of the patient and reduces the core-to-peripheral tissue temperature gradient, which otherwise promotes the heat redistribution after spinal anesthesia [18]. Intraoperative forced-air warming has been shown to be uncomfortable for the patient and may affect the early maternal-newborn bonding [3]. Unlike the forced-air warming, warmed intravenous fluids do not disturb the operation during the surgical procedure. Despite the existence of prospective studies on active warming during cesarean delivery, no consensus regarding its efficacy exists. Studies have suggested that single-modality interventions to prevent hypothermia (forced-air or intravenous fluid warmings) result in only marginal or no benefit for patients undergoing cesarean sections [1, 4, 19, 20].

Therefore, we aimed to evaluate the effect of the combined application of 30 min of preoperative warm forced-air and perioperative warm intravenous fluids in women receiving spinal anesthesia for cesarean deliveries and we assumed that combination of warmed preoperative forced-air and warmed perioperative intravenous fluids could prevent maternal hypothermia during cesarean sections under spinal anesthesia.

## Methods

### Study design

The Ethical Committee of Ningbo NO.7 Hospital approved this study, which follows the tenets of the Declaration of Helsinki, and we pre-registered it at http://www.chictr.org.cn/index.aspx (ChiCTR1800019117). This study adheres the applicable CONSORT guidelines. We enrolled healthy pregnant women undergoing elective cesarean deliveries under spinal anesthesia after obtaining their informed consents. American Society of Anesthesiologists physical status I-II parturients, aged 18 to 40 years, with more than 37-week gestations, singleton pregnancies, and scheduled for cesarean delivery under spinal anesthesia were eligible for enrollment. We excluded women with coagulation abnormalities, thyroid disease, cesarean delivery using epidural or general anesthesia, and baseline temperatures ≥37.5 °C.

### Study protocol

After obtaining the signed informed consents, we randomly allocated eligible participants to either the control or the intervention groups. Randomization was computer-generated using Microsoft Excel’s random number generator, and we concealed allocations using sequentially numbered opaque sealed envelopes.

All parturients fasted for 8 h before the cesarean section. Once in the preoperative waiting area, the parturients in the intervention group received 30 min of upper body preoperative warming using a forced-air warming device (EQ-5000 230 V, Smiths Medical ASD, Rockland, USA) set to 43 °C and nurses established intravenous accesses. The women in the intervention group received Ringer’s lactate solution pre-warmed to 37 °C through a 3MRanger™ Fluid Warmer until the end of the procedure. We monitored the patients during the interventions. We discontinued the intervention in cases in which the parturients experienced adverse side effects related to warming such as diaphoresis or nausea and vomiting, or if the core thermometer was > 37.5 °C.

After prewarming, we immediately transferred the term parturients to the operating room (OR). Participants in the intervention group received 30 min of upper body preoperative warming in the preoperative waiting area, and received IV fluid warming during the observation period (preoperative waiting area, OR and PACU). The women in the control group received usual care consisting of no active warming and they received the intravenous fluid at room temperature throughout the procedure (preoperative waiting area, OR and PACU). We recorded data on vital signs including heart rate, blood pressure, hemoglobin peripheral saturation, and baseline core temperature in the preoperative area. The same operator measured patients’ core temperatures using an infrared tympanic thermometer (PRO6000, Braun, Marlborough, MA USA 01752) with disposable covers, and recorded the average value of three measurements. The hospital maintained central control of the temperatures of the preoperative area, OR, and post-anesthesia care unit (PACU), and we obtained the temperature readings from the thermostat.

An anesthesiologist not involved in the study applied all spinal anesthesias at the L3–4 interspace, with 2 mL of 0.5% plain bupivacaine, using a 25-gauge Quincke needle. The surgeon commenced the operations once a sensory blockade above the T4 level was achieved according to the results of pinprick tests. After the operation, all patients were transferred to the PACU covered with a cotton sheet and a blanket.

We obtained values for core temperature, maternal thermal comfort scores, and the incidences of shivering and hypothermia at the following timepoints: T_0_ = baseline, T_1_ = pre-spinal, T_2_ = post-spinal, T_3_ = after 15 min in the OR, T_4_ = after 30 min in the OR, T_5_ = surgery end, T_6_ = PACU arrival, T_7_ = after 15 min in the PACU, T_8_ = after 30 min in the PACU. According to Guidelines [21], we defined maternal hypothermia as a core temperature < 36 °C. We assessed thermal comfort scores using a verbal numerical scale on which we defined 0 as completely unsatisfied with the “thermal comfort” and 100 as completely satisfied. We graded shivering during and after the cesarean section according to the Bedside Shivering Assessment Scale (0, no shivering; 1, shivering localized to the core and neck; 2, shivering including the upper extremities; 3, total body shivering) [22]. The anesthesiologist provided meperidine according to their own criteria. A midwife recorded neonatal axillary temperature, and Apgar scores at 1 and 5 min after birth. Based on our institutional guidelines, if the core temperature was lower than 35.5 °C, rescue warming would performed for the parturients by using a forced-air warming device.

We defined bradycardia as a heart rate < 50 beats/min, and treated it with 0.5 mg of intravenous atropine. When the systemic pressure decreased more than 30% of the baseline pressure or dropped below 90 mmHg, we administered ephedrine (5 mg). Mean arterial pressure and heart rate was measured at baseline, prespinal, postspinal and at the end of the procedure.

We recorded demographic data (age, height, weight, parity, and gravidity) and surgical and anesthetic variables (Preoperative and total volume of intravenous fluids, estimated blood loss, duration of surgery, and the ambient temperatures in the preoperative area, OR, and PACU).

### Statistical analyses

The primary outcome measure was the core temperature change between two groups from baseline to the end of the surgical procedure. Secondary outcomes included thermal comfort scores during the operation, the incidence of shivering and hypothermia (< 36 °C), the core temperature on the arrival at the PACU, neonatal axillary temperature at birth, and Apgar scores at 1 and 5 min).

Analysis of covariance for repeated measures was under taken to calculate the sample size. A bonferroni correction for multiple pairwise comparisons was used, giving an adjusted *P* value level of significance (*P* < 0.01). A clinically significant difference in the core temperature between study groups was set at 0.4 °C according to our pilot trial with a standard deviation of 0.5 °C,which was also consistent with Chung et al’ s study [23]. A sample size of120 patients, including 20% dropouts, was estimated to provide 90% power for detecting a statistically significant difference between groups at an α level of 0.01.

We expressed normally distributed continuous data as means ± SDs, and compared variables between study groups using the Student t test. Nonparametric data are presented as medians (interquartile ranges), and compared between study groups using the Mann–Whitney U test. We investigated associations among discrete variables using the χ2 or Fisher exact tests. Two-way repeated measures ANOVA was applied with change from baseline as the dependent variable, and the intervention, time, and the treatment multiplied by time interaction as independent variables. We also used two-way repeated measures ANOVA to assess the core temperature change and the thermal comfort between groups at each timepoint. We performed all statistical analyses using the SPSS software (version 22.0, SPSS, Chicago, IL, USA). We considered *P*-values < 0.05 as statistically significant.

## Results

**Fig. 1:**
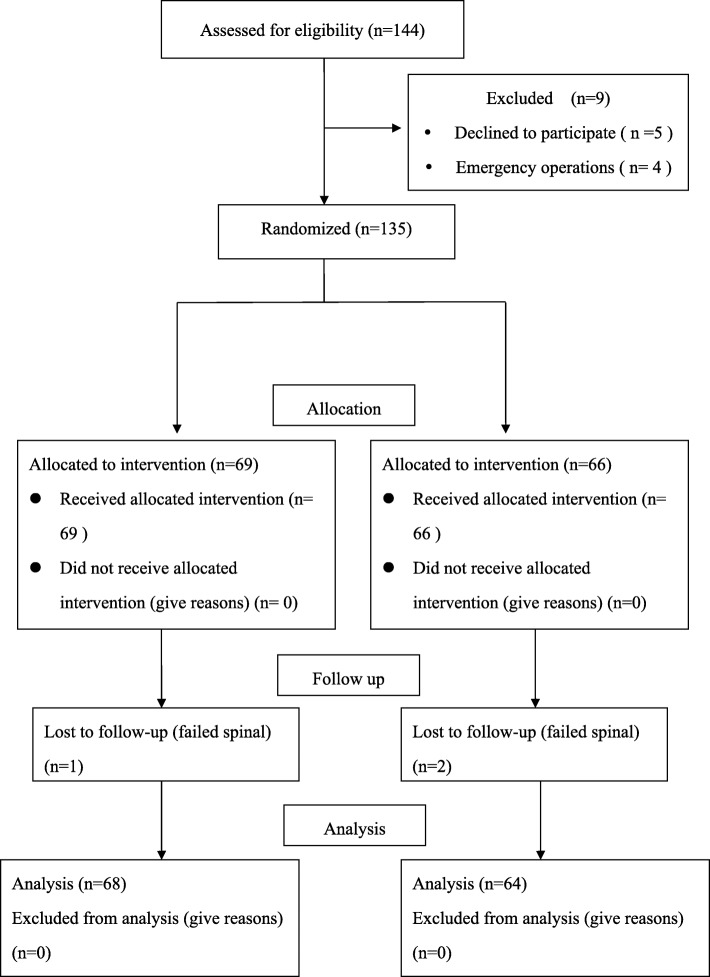
Flow diagram outlining the enrollment and randomization study procedures

Patients were enrolled in the study between January 2019 and June 2019.We considered 144 patients for eligibility, and excluded 9 before randomization. In the end we randomly allocated 135 patients to one of the two groups (69 women to the intervention group, and 66 to the control group). We had to exclude one patient from the intervention group and two patients from the control group due to failed spinal anesthesia (Fig. 1). The demographic and obstetric characteristics, as well as the surgical and anesthetic values, were did not differ significantly between the two groups. Vital sign parameters such as peripheral oxygen saturation, heart rate, mean arterial pressure measurement at the each point, and the incidence of hypotension and vomiting, ephedrine dose administered also had no difference between two groups during the observation period. The room temperatures in the preoperative area, OR, and PACU were similar for the two groups (Table 1).

**Table 1:** Demographic, surgical, and anesthetic characteristics of the study population

	Intervention(*n* = 68)	Control(*n* = 64)
Age, y	28.5 ± 5.1	27.5 ± 4.6
BMI, kg/m^2^	28.1 ± 3.2	28.4 ± 3.4
Gestation, weeks	38.6 ± 1.4	38.7 ± 1.3
Gravidity	2.0 [1.0–3.5]	2.0 [1.0–3.0]
Parity	1.0 [0.0–1.0]	1.0 [0.0–1.0]
ASA I, n(%)	56(82.4%)	52(81.3%)
Hypotension, n(%)	30(44.1%)	28(43.8%)
Ephedrine,mg	13.6 ± 9.7	12.5 ± 8.5
Vomiting, n(%)	7(10.3%)	6(9.4%)
Estimated blood loss, mL	272 ± 70	267 ± 74
Exposed time, min duration,min	50.2 ± 7.7	48.9 ± 8.1
Baseline temperature, °C	36.7 ± 0.4	36.7 ± 0.3
Prespinal temperature, °C	37.0 ± 0.3	36.6 ± 0.3
Preoperative ambient temperature, °C	22.6 ± 0.6	22.9 ± 0.8
OR ambient temperature, °C	23.9 ± 0.7	24.2 ± 0.8
PACU ambient temperature, °C	23.2 ± 0.6	22.8 ± 0.6
Preoperative crystalloid amount, mL crystalloid amount	371 ± 55	363 ± 55
Total crystalloid amount, mL	1291 ± 246	1343 ± 264

Data are expressed as means ± SDs, medians [interquartile ranges], or numbers (%)

Exposed time: including surgery duration, clean up time, and finding sheets time

*BMI* body mass index, *ASA* American Society of Anesthesiologists, *OR* operating room, *PACU* post-anesthesia care unit

**Fig. 2:**
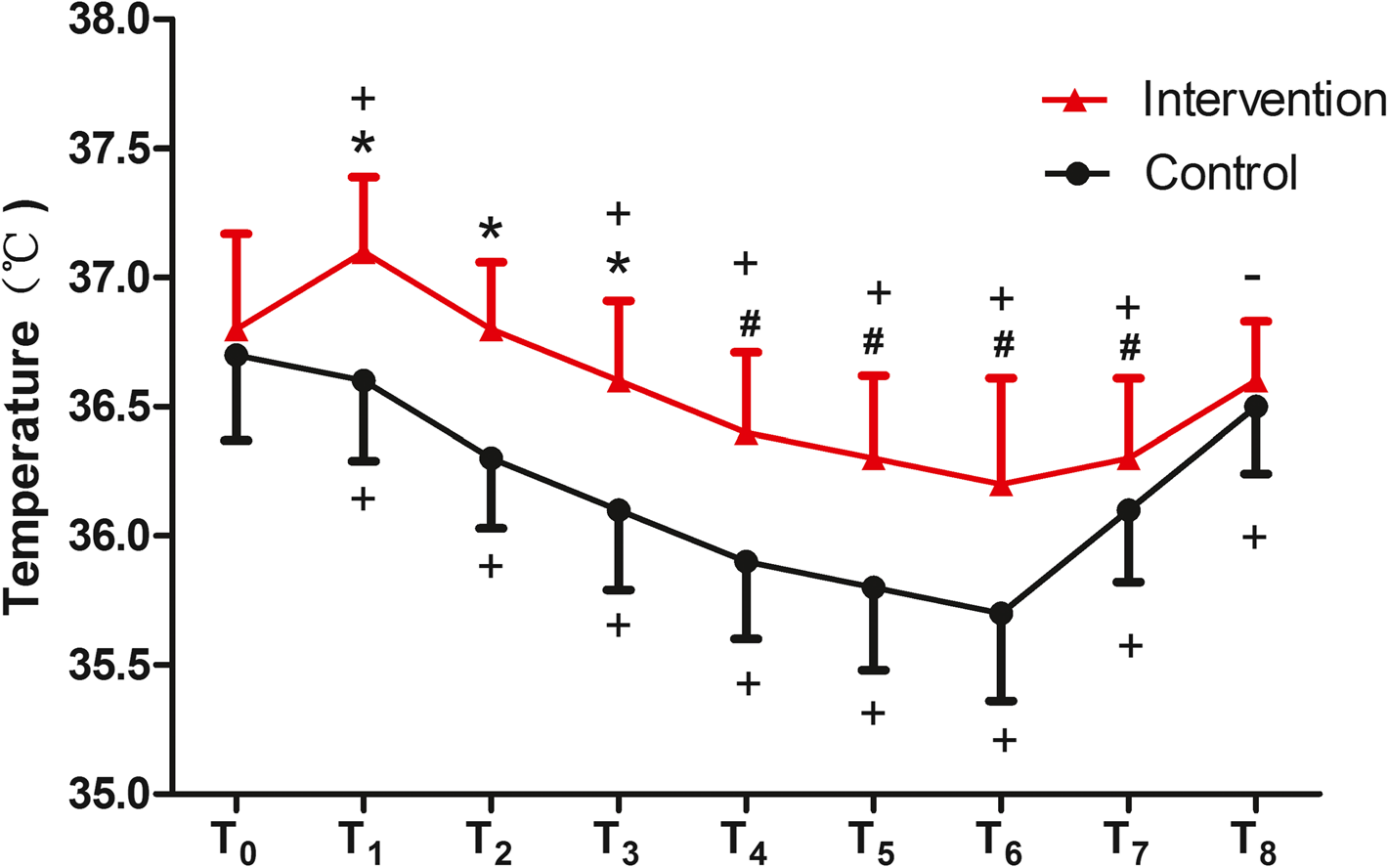
Core tympanic temperatures during the procedure. Compared with control group, the patients in intervention group experienced higher perioperative mean temperatures during the procedure (T_1_-T_3_, *P* < 0.001, T_4_-T_7_, *P* < 0.05). Timepoints: T_0_ = baseline, T_1_ = pre-spinal, T_2_ = post-spinal, T_3_ = after 15 min in the OR, T_4_ = after 30 min in the OR, T_5_ = surgery end, T_6_ = PACU arrival, T_7_ = after 15 min in the PACU, T_8_ = after 30 min in the PACU. OR: operating room; PACU: post-anesthesia care unit. *P < 0.001,^#^*P* < 0.05 refer to statistically significant differences between the intervention and the control groups. ^+^*P* < 0.001_,_^−^*P* < 0.05 refer to comparisons with the baseline (T_0_)

Our two-way repeated measures ANOVA analysis revealed a significant difference in the core temperature changes from the T_1_ to T_7 _timepoints between the two groups (*F* = 13.022, *P* < 0.001), and the group×time interaction difference was also significant (*F* = 23.195, *P* < 0.001). The patients in the intervention group experienced higher perioperative mean temperatures during the procedure than those in the control group (T_1_-T_3_, *P* < 0.001, T_4_-T_7_, *P* < 0.05). In the control group, the core temperature was decreased at all the time points compared to the baseline. We also found a slight decline in the core temperatures from the baseline during the procedure (except T_1_and T_2_) in the intervention group (Fig. 2).

**Fig. 3:**
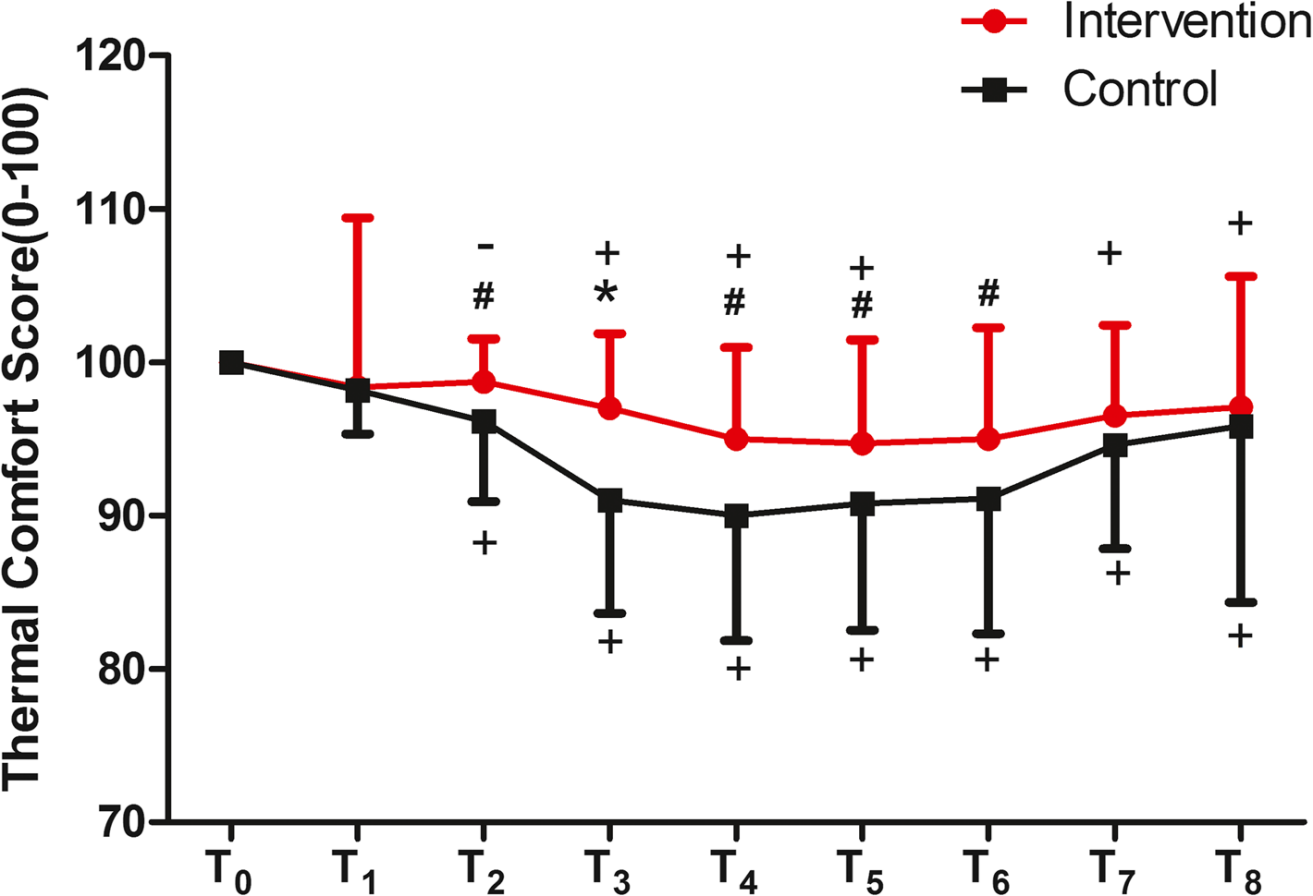
Maternal comfort scores during the procedure. The maternal thermal comfort scores differed significantly between two groups from the T_2_ to T_6_ timepoints (all *P* < 0.05 or *P* < 0.001). Timepoints: T_0_ = baseline, T_1_ = pre-spinal, T_2_ = post-spinal, T_3_ = after 15 min in the OR, T_4_ = after 30 min in the OR, T_5_ = surgery end, T_6_ = PACU arrival, T_7_ = after 15 min in the PACU,T_8_ = after 30 min in the PACU. We measured thermal comfort scores using a verbal numerical scale in which 0 was defined as completely unsatisfied with their “thermal comfort” and 100 as completely satisfied. *P < 0.001,^#^*P* < 0.05 refer to statistically significant difference between the intervention and the control groups. ^+^*P* < 0.001_,_^−^*P* < 0.05 refer to comparisons with baseline (T_0_)

Thermal comfort scores were higher in the intervention group than in the control group (*F* = 9.847, *P* = 0.002*),* the group × time interaction difference was also significant (*F* = 2.750, *P* = 0.008). The maternal thermal comfort scores differed significantly between two groups from the T_2_ to T_6_ timepoints (all *P* < 0.05 or *P* < 0.001). In comparisons with the baseline thermal comfort scores, the timepoints in the control group (except T_1_) and those in the intervention group (except T_1_and T_6_) all exhibited decreased thermal comfort scores (Fig. 3).

Core temperatures on arrival at the PACU were greater in the intervention group (36.2 ± 0.4 °C) than in the control group (35.5 ± 0.3 °C), *P* = 0.007. The incidences of shivering were 56.3% in the control group and 19.1% in the intervention group during the surgical procedure (*P* < 0.001), and the shivering assessment scores were higher in the control than in the intervention group. The overall incidence of perioperative hypothermia was significantly lower in the intervention group than in the control group (*P* < 0.001). Neonatal outcomes were similar between the two groups (Table 2).

**Table 2:** Secondary maternal and neonatal outcomes

	Intervention (n = 68)	Control (n = 64)	*P* value
PACU arrival temperature °C	36.2 ± 0.4	35.5 ± 0.3	0.007
Delivery temperature °C	36.6 ± 0.3	36.0 ± 0.3	< 0.0001
Perioperative shivering	13(19.1%)	36(56.3%)	< 0.0001
Shivering assessment score	0 [0–0]	1 [0–2]	0.01
Perioperative hyperthermia	14(20.6%)	33(51.6%)	< 0.0001
Apgar score			
1 min	9 [9–9]	9 [9–9]	0.21
5 min	10 [10–10]	10 [10–10]	0.43
Neonatal temperature °C	36.3 ± 0.3	36.3 ± 0.4	0.350

Data are expressed as means ± SDs, medians [interquartile ranges], or numbers (%)

Bedside Shivering Assessment Scale: 0 = no shivering; 1 = shivering localized to the core and neck; 2 = shivering including the upper extremities; 3 = total body shivering

*OR* operating room, *PACU* post-anesthesia care unit

## Discussion

In our study, our intervention with 30 min preoperative forced-air warming and perioperative administration of warmed intravenous fluids reduced the extent of core temperature decline, decreased the incidence of preoperative hypothermia and shivering, and improved maternal comfort in patients undergoing cesarean section with spinal anesthesia as opposed to the outcomes in the control group patients.

The results of our study are similar to those of the study by Chung et al. in which preoperative forced-air warming prevented hypothermia and shivering in patients undergoing elective cesarean delivery with spinal anesthesia [23]. However, in that study the difference in maternal temperatures between groups was evident only at one timepoint (45 min after prewarming). Therefore, the impact of their single intervention was likely smaller than the impact of our combined warming of forced-air and intravenous fluids. The combined active warming modalities applied in our intervention group maintained a significantly higher mean temperature nearly throughout the entire surgical procedure (at the seven timepoints). The intervention group had a significantly higher temperature on arrival at the PACU compared with the control group. Similarly, our study also demonstrated that our combined warming technique can reduce the incidence of perioperative hypothermia significantly (20.6% in the intervention group compared with 56.3% in the control group).

Unlike forced air-warming which warms the patient from the outside, the warming of intravenous fluids prevents hypothermia by offsetting the 0.25°C reduction in body temperature that occurs with each liter of intravenous fluids administered at room temperature [24]. To minimize spinal hypotension, women undergoing caesarean delivery often receive large volumes of intravenous fluid intraoperatively. Thus, fluid warming may be particularly important and effective during caesarean deliveries [25]. These findings agree with those in other studies. Horn et al. found that 15 min of preoperative warming provided additional efficacy when added to warmed intravenous fluids in the setting of epidural anesthesia, resulting in an average 1°Cdifference between control and intervention groups at the end of the operations [13]. Unlike forced air-warming during all the surgical procedure [25], brief period of prewarming, would be more acceptable to awake patients, easy to accommodate and could be combined with intraoperative warming, which is undoubtedly effective once the redistribution period has passed.

The combined technique has the potential to minimize maternal temperature drops. Similarly, in a study by De Bernardiset al, thermal gowns and warmed intravenous fluids decreased the patient temperature drops and the incidence of shivering as compared to the same variables in the control group [26]. In contrast, in Munday et al’s study, 20 min of preoperative forced-air warming with intravenous fluid warming did not prevent temperature drops in women undergoing cesarean delivery [27]. However, the OR ambient temperature was lower in that study (21.4 °C). The time between the end of the warming regime and the OR entry was longer than those in our study. In their study, the time interval was smaller than 20 min, but some women may have experienced longer delays. Therefore, their study design may have been less powerful than ours to detect differences between two groups.

In our study, shivering was significantly less common in the patients who were actively warmed, a finding that may be explained by the significantly higher core temperatures in the combined active warming patients than in the controls. The intensity and incidence of shivering may indicate the severity of hypothermia. Our study showed that the overall incidence of perioperative hypothermia decreased significantly in the intervention group compared to the incidence in the control group. Shivering is both thermogenic accompanied by vasoconstriction or non-thermogenic as that induced by catecholamines resulting from pain or anxiety [28]. A meta-analysis demonstrated that warmed intravenous fluids are effective at reducing the incidence of hypothermia and shivering [29]. In addition, our combined active warming interventions improved the thermal comfort scores of the patients in the intervention group as opposed to the score in the control group. Thermal comfort scores are subjective measures of patient comfort during the perioperative period, and may differ from actual temperature measurements and do not necessarily reflect recorded shivering episodes. Results of studies [20, 30, 31] and a meta-analysis [29] suggest that forced-air warming can improve thermal comfort scores.

We found no significant differences in neonatal outcomes between the two groups, which is not surprising given our small sample size and our limited neonatal outcome measurements. Though our study found that the patients in the intervention group experienced higher temperatures at the time of delivery, but both groups all had normal core temperatures with a difference of 0.6 degrees, which did not affect the neonatal temperature. Further studies specifically powered to evaluate the impact of active warming on neonatal outcomes are still required.

We are aware of the limitations in our study. Our infrared tympanic thermometers lack evidence of their quality and accuracy. However, they are not invasive and provide an acceptable and comfortable measurement to patients. Also, we did not use intrathecal morphine as a spinal anesthetic. However, many institutions prefer to use intrathecal opioids for postoperative analgesia after cesarean delivery, so this may affect the generalizability of our study. A study has shown that intrathecal morphine administration may exacerbate hypothermia [19]. Finally, it was not a blinding clinical trial, and it may increase the bias.

## Conclusion

In all, preoperative forced air-warming combined with perioperative intravenous fluid warming may prevent maternal hypothermia, reduce maternal shivering, and improve maternal thermal comfort in those undergoing cesarean section with spinal anesthesia.

## Data Availability

The datasets used and/or analyzed during the current study are available from the corresponding author on reasonable request.

## Abbreviations

ASA: American Society of Anesthesiologists
BMI: Body mass index
OR: Operating room
PACU: Post-anesthesia care unit
